# Real-Time PCR Assay for the Diagnosis and Quantification of Co-infections by *Diaporthe batatas* and *Diaporthe destruens* in Sweet Potato

**DOI:** 10.3389/fpls.2021.694053

**Published:** 2021-06-22

**Authors:** Kazuki Fujiwara, Yuki O. Kobayashi, Manami Usui, Kazuya Nishioka, Misa Nakamura, Shinji Kawano, Yoshihiro Okada, Akira Kobayashi, Atsushi Miyasaka, Kazuyuki Hirayae, Yoshiyuki Kushima, Yatsuka Nishi, Hiroyoshi Inoue

**Affiliations:** ^1^Institute for Plant Protection, National Agriculture and Food Research Organization (NARO), Tsukuba, Japan; ^2^Division of Agro-Environment Research, Kyushu Okinawa Agricultural Research Center, NARO, Koshi, Japan; ^3^Division of Upland Farming Research, Kyushu Okinawa Agricultural Research Center, NARO, Miyakonojo, Japan; ^4^Miyazaki Agricultural Experiment Station, Miyazaki, Japan; ^5^Kagoshima Prefectural Institute for Agricultural Development, Kagoshima, Japan; ^6^Okinawa Prefectural Agricultural Research Center, Itoman, Japan; ^7^Division of Agro-Environment Research, Kyushu Okinawa Agricultural Research Center, NARO, Itoman, Japan

**Keywords:** *Diaporthe batatas*, *Diaporthe destruens*, foot rot disease, real-time PCR, sweet potato

## Abstract

Foot rot disease caused by *Diaporthe destruens* (formerly *Plenodomus destruens*) has become a major concern for the production of sweet potato [*Ipomoea batatas* (L.) Lam.] in Japan. A related fungus *Diaporthe batatas*, which causes dry rot disease of sweet potato, is native and is widespread in fields in Japan. The similar characteristics of these two pathogens pose a challenge for conventional disease diagnosis. Currently, there are no effective molecular measures for identifying and distinguishing *D. destruens* and *D. batatas*. Here, we demonstrate a real-time PCR assay that distinguishes and quantifies *D. batatas* and *D. destruens* from co-infected sweet potato. The assay was performed with various simulated DNA combinations of *D. batatas* and *D. destruens* ranging from 1:1 to 1:100000. The assay was also used with the ratios of *D. batatas*: *D. destruens*: sweet potato DNA ranging from 1:1:1 to 1:1:100000. These assays produced a specific amplification product for each of the pathogens, and quantified the fungal biomass over the entire range tested without detecting false positives. The assay was validated by using infected sweet potato collected from various fields; it showed sufficient sensitivity and specificity to quantify and distinguish *D. batatas* and *D. destruens* from these field samples. Thus, our real-time PCR assay would be a useful tool for diagnosis of *D. batatas* and *D. destruens* and is expected to provide the foundation for the design of integrated disease management strategies for foot rot disease in sweet potato.

## Introduction

Foot rot is an important fungal disease in sweet potato [*Ipomoea batatas* (L.) Lam.]. The disease was first observed in Virginia, United States (Harter,1913a,b; Harter and Weimer, 1929) and is widespread in some countries in North and South America (Lopes et al., 1994; Clark et al., 2013), eastern Africa (Steinbauer and Kushman, 1971; Skoglund and Smit, 1994), Asia (Huang et al., 2012; Gai et al., 2016; Paul et al., 2019), and New Zealand (Liu et al., 2014). The causal pathogen is *Diaporthe destruens* (Harter) Hirooka, Minosh, and Rossman [formerly *Plenodomus destruens* Harter; syn. *Phomopsis destruens* (Harter) Boerema, Loerakker, and Hamers). Foot rot infection in sweet potato causes black stem, wilting, and plant death due to stem girdling. Root rot is often developed in storage roots. Severe infection involves hardening of the black stem lesion and rotten tubers in the soil. In Japan, foot rot disease was first observed in Southern Kyushu and Okinawa regions (Kobayashi, 2019) including Okinawa and Kagoshima Prefectures in 2018, and Miyazaki Prefecture in 2019. In sweet potato cultivation, development of foot rot disease is often indistinguishable from that of other soil-borne diseases including dry rot disease caused by *Diaporthe batatas* Harter & E.C. Field (Udayanga et al., 2015), Fusarium wilt of sweet potato caused by several *Fusarium* spp. such *F. acuminatum, F. incarnatum, F. oxysporum*, *F. proliferatum*, and *F. solani* (Scruggs and Quesada-Ocampo, 2016), and bacterial wilt and root rot of sweet potato caused by *Dicheya* spp. (syn. *Erwinia chrysanthemi*; Schaad and Brenner, 1977). The complex of symptoms often masks the emergence of foot rot disease. Of the above pathogens, *D. batatas* rarely causes serious damage to sweet potatoes in the field; however, even if the storage roots look healthy at harvesting, the infection triggers further disease development, similar to that caused by *D. destruens*, in the roots during storage. Thus, whereas foot rot disease manifests in the field, the damage increases post-harvesting in cases of co-infection with *D. batatas* and *D. destruens.* This co-infection makes it more difficult to identify a causative pathogen, and hinders the preparation of seed tubers for planting in the following year. Thus, we need a novel molecular measure to effectively identify and distinguish *D. batatas* and *D. destruens*.

Molecular methods for rapid detection and identification of causative pathogens are powerful tools in disease diagnosis. The internal transcribed spacer (ITS) regions of ribosomal DNA (rDNA) are commonly used in the identification and phylogenetic study of fungi (Chase and Fay, 2009; Seifert, 2009). PCR-based methods using the ITS sequences have been successfully used to identify *Diaporthe* spp. and *Phomopsis* spp. (Baumgartner et al., 2013; Udayanga et al., 2014), which are difficult to discriminate by morphological features alone (Hosseini et al., 2020). Currently, conventional PCR (Lin et al., 2017) and loop-mediated isothermal amplification (LAMP; Maruyama et al., 2020) are the only techniques available for diagnosis of *D. destruens*, but there are technical concerns about their sensitivity and specificity for assessing infection with *D. destruens*. There is currently no robust method for disease management of *D. destruens*, and the disease management relies on pathogen diagnostic measures to determine the cause of disease and degree of infection for informing the decision-making in countermeasures. Therefore, development of an efficient molecular diagnostic tool for foot rot disease is essential. To this end, we have established a real-time PCR technique for reliable evaluation of both *D. batatas* and *D. destruens* in co-infected sweet potato tissues.

## Materials and Methods

### Pathogens Used in This Study

Sweet potato seedlings and storage roots with appearance of foot rot symptoms were collected from several local fields in the Miyazaki, Kagoshima, and Okinawa Prefectures, Japan, in 2018. Infected plant materials were washed with sterilized distilled water, and 1-cm^3^ pieces of the materials were cut and plated onto potato dextrose agar (PDA) plates (BD, NJ, United States) with 50 μg mL^–1^ of ampicillin (Sigma, Munich, Germany) or sweet potato dextrose agar (SPDA) plates [50% sweet potato filtrate obtained by boiling 500 *g* diced sweet potato in 1,000 mL distilled water and filtering, 20 *g* glucose (FUJIFILM Wako, Tokyo, Japan), and 15 *g* agar (FUJIFILM Wako, Tokyo, Japan)]. After 2 weeks, small fungal colonies were picked with a sterilized toothpick and transferred to fresh PDA or SPDA plates. Fungal colonies were subjected to *Diaporthe* species identification based on morphological characteristics and DNA sequencing. Single spores of *D. destruens* and *D. batatas* were isolated under a light microscope, and isolates were cultured as described above, providing 11 *D. batatas* isolates and 17 *D. destruens* isolates (Supplementary Table 1). We also used the following *Diaporthe* and *Phomopsis* species provided from the National Agriculture and Food Research Organization (NARO) Genebank^1^ : *D. gardeniae* (MAFF 410870), *D. nobilis* (MAFF 245916), *D. santonensis* (MAFF 410114), *P. asparagi* (MAFF 150068), *P. cucurbitae* (MAFF 410452), *P. fukushii* (MAFF 625033), *P. macrospora* (MAFF 410314), *P. velata* (MAFF 410118), and *P. vexans* (MAFF 150147). These fungal species were maintained on PDA or SPDA plates until use. To prepare culture plate samples for DNA extraction, single fungal disks were taken from the culture plates by a 5-mm cork borer, and then suspended in AP1 buffer [a component of the DNeasy Plant Mini Kit (Qiagen, Venlo, Netherlands)]. The samples were then stored at −20°C until use.

Isolates of *D. destruens* were cultured on SPDA plates at 25°C in dark for 5 days and then exposed to black light radiation for 2 weeks by using an FL20S black light (Toshiba, Tokyo, Japan) to induce sporulation. Alpha-conidia were collected from the culture plates and suspended in sterilized distilled water. The conidia were adjusted to a density of ∼1 × 10^7^ cells mL^–1^ by using a hemocytometer under a light microscope. Collection of *D. batatas* conidia was performed as described for *D. destruens* conidia. The conidial suspensions were stored at −20°C until use.

### DNA Extraction

All prepared samples were disrupted in a Multi-Bead Shocker (Yasui Kikai, Osaka, Japan) with 1 mm diameter zirconia beads (Sarstedt, Nümbrecht, Germany) at 3,000 rpm for 45 s, and the disrupted samples were chilled on ice for 3 min. This procedure was performed three times. DNA from conidial suspensions, culture plates, and plant materials were extracted with a DNeasy Plant Mini Kit or DNeasy Plant Maxi Kit (Qiagen, Venlo, Netherlands) according to the manufacturer’s instructions. In conidial and culture plate samples, three and five biological replicates, respectively, were used to prepare DNA templates. Each of the DNA templates was adjusted to 5 ng μL^–1^. The DNA templates from conidial samples were used to prepare a 10-fold dilution series of DNA from 5 to 0.00005 ng μL^–1^.

To assess detection efficiency in co-infection of *D. batatas* and *D. destruens*, a fixed concentration of fungal DNA of one pathogen (5 ng μL^–1^) was mixed with a progressively decreasing DNA concentration of the other pathogen (5, 0.5, 0.05, 0.005, 0.0005, and 0.00005 ng μL^–1^). This provided a ratio of DNA concentrations in the range from 1:1 down to 1:100000. To simulate co-infection with sweet potato, an increasing concentration of sweet potato DNA (5 to 50 ng μL^–1^) was mixed with a decreasing concentration of *D. batatas* and *D. destruens* DNA (5 to 0.0005 ng μL^–1^ each). This generated a ratio of DNA concentrations of *D. batatas*: *D. destruens*: sweet potato from 1:1:1 to 1:1:100000. For preparation of DNA templates, *D. batatas* strain MOKM-3S-B and *D. destruens* strain KTJ-1R-a were used as representative strains. A sweet potato DNA template was prepared from healthy tubers.

### Development and Specificity of Primers

We designed new primer sets Db ITS and Dd ITS (Table 1) from ITS1 and ITS2 of rDNA by comparison of the DNA sequences of *D. batatas* (KU577616, MG827239, and NR_152456) and *D. destruens* (JN848791, MH465671, MH465672, and MH465673) obtained from the NCBI database and the DNA sequences of 11 *D. batatas* isolates and 17 *D. destruens* isolates. The primer design was carried out using Primer-BLAST^2^.

**TABLE 1:** Primers used in this study.

Primers	Sequences (5′–3′)	References
Db ITS-F	GTTTCTATAGTGAATCTCTGAGT	This study
Db ITS-R	TCCAGAGCGAGATGTAACTA	This study
Dd ITS-F	GTTTTTATAGTGTATCTCTGAGC	This study
Dd ITS-R	GGCCTGCCCCCTTAAAAA	This study
SPPD3f	TCTCTGCTGAGGCCCCCCGGAGA	Lin et al., 2017
SPPD3r	AAGGCAGTGCCCCATCACCAAGCCAG	Lin et al., 2017
ITS1	TCCGTAGGTGAACCTGCGG	White et al., 1990
ITS4	TCCTCCGCTTATTGATATGC	White et al., 1990

Primer specificity was tested in both conventional PCR and real-time PCR. Db ITS and Dd ITS primers were tested against closely related *Diaporthe* species and *Phomopsis* species as well as *D. batatas* and *D. destruens*. Template DNA was prepared from culture plates of each fungal species as described above. In conventional PCR, a single PCR mixture was prepared by mixing three biological replicates of each of fungal pathogen DNA (5 ng μL^–1^). Real-time PCR reactions were prepared from three biological replicates of each of fungal pathogen DNA.

### Conventional PCR and Real-Time PCR Conditions

For conventional PCR with Db ITS or Dd ITS primer sets, we used TaKaRa Ex Taq Hot Start Version (Takara, Tokyo, Japan) according to the manufacturer’s instructions. Reaction mixtures contained 0.1 μL of *TaKaRa Ex Taq* HS, 2 μL of 10 × *Ex Taq* Buffer, 1.6 μL of dNTP Mixture, 0.5 μM of each primer (Forward/Reverse), and 2 μL of DNA template in a final volume of 20 μL. The conventional PCR protocol consisted of initial denaturation at 94°C for 2 min, and 32 cycles of denaturation at 98°C for 10 s, annealing at 55°C for 30 s, and extension at 72°C for 1 min. The PCR products were detected by 1% agarose gel electrophoresis. We also used a SPPD primer set (Table 1) for conventional PCR as previously reported (Lin et al., 2017); these PCR products were also detected by 1% agarose gel electrophoresis.

Real-time PCR was performed in a QuantStudio 5 real-time PCR system (Thermo Fisher Scientific, MA, United States) using SYBR Premix ExTaq II (Takara, Tokyo, Japan) according to the manufacturer’s protocol. Reaction mixtures contained 1 × TB Green Premix Ex Taq II, 1 × ROX Reference Dye II, 0.4 μM of each primer (Forward/Reverse), and 2 μL of DNA template in a final volume of 20 μL. The real-time PCR protocol consisted of initial denaturation at 94°C for 1 min, and 32 cycles of denaturation at 96°C for 30 s, annealing at 55°C for 30 s, and extension at 72°C for 30 s. Further denaturation at 96°C for 15 s, holding at 55°C for 1 min, and heating from 55 to 96°C for 15 s were carried out for melting curve analysis. In both conventional and real-time PCR, stem and tuberous DNA of healthy sweet potato plants as well as no template were used as negative controls.

### Validation of Real-Time PCR Assay

The simulation of standard curve using a 10-fold dilution series of DNA from 5 to 0.00005 ng μL^–1^ was carried out. The regression equations and *R*^2^ coefficient of determination for quantification cycle (Cq) versus the natural logarithm of concentration of amplified genomic DNA (gDNA) were calculated. The amplification efficiency (%) was calculated by the following equation: [10^(–1/(slope))^ − 1] × 100 based on logarithm of gDNA concentration.

### DNA Sequencing

Fungal isolates were subjected to DNA sequencing analysis. Culture plates of each isolate were used for DNA extraction as described above. Conventional PCR was performed using a universal ITS1 and ITS4 primer set (White et al., 1990; Table 1), which amplifies the ITS regions in fungi; the PCR protocol was initial denaturation at 95°C for 4 min, followed by 35 cycles of denaturation at 95°C for 1 min, annealing at 56°C for 1 min, and extension at 72°C for 2 min, with a final extension for 10 min at 72°C. Each conventional PCR product was purified with the NucleoSpin Gel and PCR Clean-up (Takara, Tokyo, Japan). DNA sequencing was performed in an ABI PRISM 3100-Avant Genetic Analyzer (Thermo Fisher Scientific, MA, United States) using the BigDye Terminator v3.1 Cycle Sequencing Kit (Thermo Fisher Scientific, MA, United States). Unincorporated dye terminators were removed with NucleoSEQ (Takara, Tokyo, Japan). Sequences were analyzed by BLAST^3^.

### Collection of Sweet Potato Seedlings in the Field

Sweet potato plants were collected from 37 sites in the Kyushu and Okinawa regions where foot rot disease appeared under natural conditions: two from Fukuoka Prefecture, 17 from Kagoshima Prefecture, four from Kumamoto Prefecture, three from Nagasaki Prefecture, six from Miyazaki Prefecture, and five from Okinawa Prefecture. Stems and tubers with or without symptoms were harvested from June to October in 2019 and 2020. In total, 55 seedlings and 35 tubers from the above regions were obtained. We generated 77 samples from stems and 83 samples from tubers of the obtained plants. For processing, stems and tubers were washed with distilled water, and their surfaces were sterilized by wiping with a Kimwipe paper soaked with 70% ethanol before use. The stem tissues were cut into small pieces of ∼5-mm width. The tubers were cut into 1-cm^3^ pieces including the skin tissue. Each processed sample was kept in a separate plastic bag. For DNA extraction, 50 mg of plant material was ground in AP1 buffer with a mortar and pestle. The collected samples and homogenized samples were stored at −20°C until use. For both stem and tubers, the diagnostic index was scored on a scale of 0 to 2 by real-time PCR detection using the primer sets Db ITS and Dd ITS (0, no symptoms with no pathogen detected; 1, symptomless but pathogen detected; and 2, symptomatic with pathogen detected).

### Statistical Analysis

The differences in relative frequency of pathogen detection among symptomless and symptomatic sweet potato plants were assessed by the Fisher’s exact test. The differences in pathogen concentration measured as Cq value among various categories in the diagnostic index were analyzed by Steel–Dwass test. Statistical analysis was performed with R v. 3.5.0 software, and *P* values < 0.01 were classified as statistically significant.

## Results

### Primer Specificity and Validation of Real-Time PCR

**FIGURE 1:**
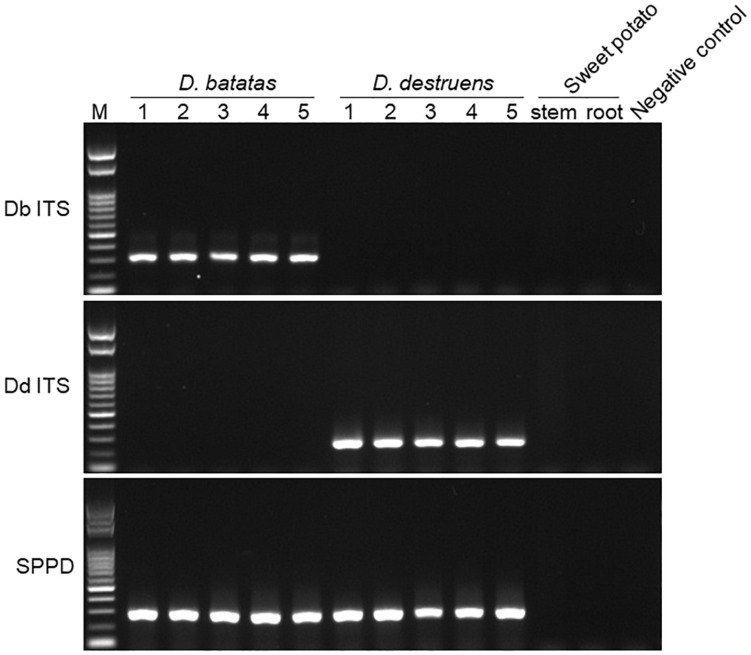
Amplification of *Diaporthe batatas* and *Diaporthe destruens* DNA by conventional PCR. Newly developed primer sets Db ITS and Dd ITS were tested against *D. batatas* and *D. destruens* DNA, respectively, in five individual PCR amplifications. The SPPD primer set previously reported was used for comparison. Template DNA (5 ng μL^–1^) were prepared from culture plates of *D. batatas* strain MOKM-3S-B and *D. destruen*s strain KTJ-1R-a, respectively. Stem and tuberous DNA of healthy sweet potato plants as well as no template were used as negative controls. Amplicons produced by Db ITS, Dd ITS, and SPPD primer sets were 317, 258, and 289 bp, respectively. M, marker.

**FIGURE 2:**
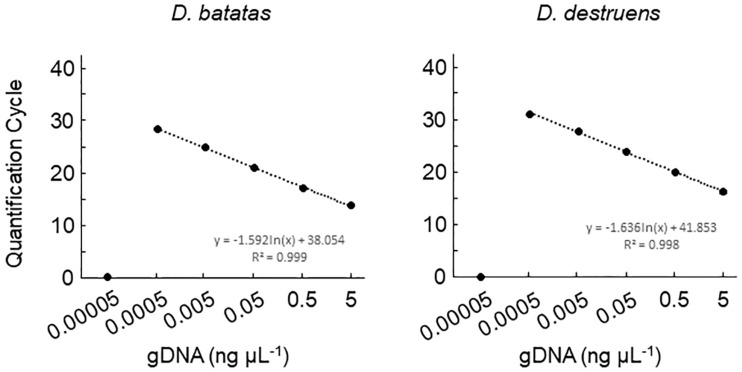
Amplification of *Diaporthe batatas* and *Diaporthe destruens* DNA by real-time PCR with Db ITS and Dd ITS primers. Template DNA was prepared from conidial samples separately from *D. batatas* strain MOKM-3S-B and *D. destruen*s strain KTJ-1R-a, and were used to prepare 10-fold DNA dilution series of DNA (5.0, 0.5, 0.05, 0.005, 0.0005, and 0.00005 ng μL^–1^). Three biological replicates of each dilution were prepared. The regression equations and *R*^2^ coefficient of determination for Cq value versus the natural logarithm of concentration of amplified gDNA are shown on the chart. Data represent means ± standard deviation where visible.

We tested the primer specificity against *D. batatas* and *D. destruens* in conventional PCR and real-time PCR. Our newly developed primers for *D. batatas* and *D. destruens* (Supplementary Figure 1) yielded different products for each pathogen in conventional PCR, i.e., a 317 bp fragment of Db ITS and a 258 bp fragment of Dd ITS (Figure 1). Conventional PCR amplifications with Db ITS and Dd ITS primers were successful for conidial and culture plate DNA of all *D. batatas* and *D. destruens* isolates that we obtained (K. Fujiwara, unpublished data). In contrast, the previously reported SPPD primer set (Lin et al., 2017) did not distinguish *D. batatas* from *D. destruens* (Figure 1). Among the tested species of the family *Diaporthaceae*, the Db ITS and Dd ITS primers were specific to *D. batatas* and *D. destruens*, respectively (Supplementary Figure 2). In real-time PCR, both Db ITS and Dd ITS primers yielded amplicons specific to *D. batatas* and *D. destruens*, respectively (Supplementary Figure 3). A single melting temperature (Tm) peak indicated that *D. batatas–*specific amplicons were detected at 89.3 (±0.4) Tm value by Db ITS and *D. destruens–*specific amplicons were detected at 86.9 (±0.4) Tm value by Dd ITS. Amplification of a 10-fold DNA dilution series (5 to 0.00005 ng DNA μL^–1^) showed a linear relationship between DNA concentration and Cq value for both Db ITS and Dd ITS primers, but the quantification was limited to concentrations of <0.0005 ng μL^–1^ in both pathogens (Figure 2). In the detection of *D. batatas*, Db ITS produced specific amplicons from 13.7 (±0.2) to 28.2 (±0.2) cycles, and Dd ITS produced specific amplicons from 16.6 (±0.3) to 31.4 (±0.1) cycles in the dilution series. The amplification efficiency was found to be 87.4% in Db ITS and 84.3% in Dd ITS. Both primers produced no false positives.

### Detection Efficiencies of *D. batatas* and *D. destruens* DNA in a Simulated Co-infection

**FIGURE 3:**
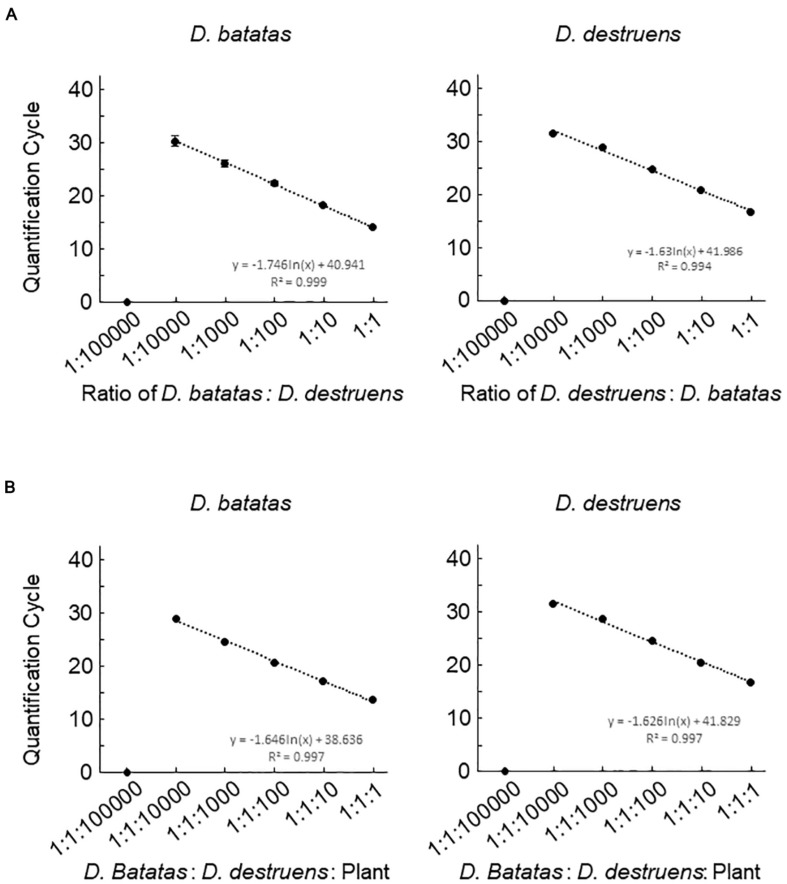
Detection efficiencies of the assay for *Diaporthe batatas* and *Diaporthe destruens* DNA in a simulated co-infection. Coinfection was simulated using mixtures of template DNA at various ratios. **(A)** mixtures of *D. batatas* and *D. destruens* DNA and **(B)** mixtures of fungal DNA and background sweet potato DNA. In the left panel of **(A)**, *D. destruens* DNA (5 ng μL^–1^) was mixed with a 10-fold dilution series of *D. batatas* DNA (5, 0.5, 0.05, 0.005, 0.0005, and 0.00005 ng DNA μL^–1^), providing ratios of *D. batatas* to *D. destruens* DNA in the range of 1:1 to 1:100000. In the right panel of **(A)** *D. batatas* and *D. destruens* were exchanged and tested at the same DNA mixing ratios. In **(B)**, ratios of *D. batatas*: *D. destruens*: sweet potato DNA from 1:1:1 to 1:1:100000 were prepared by decreasing the concentrations of *D. batatas* and *D. destruens* DNA (5 to 0.0005 ng μL^–1^) while increasing the sweet potato DNA concentration (5 to 50 ng μL^–1^). The DNA concentrations in each reaction were 5:5:5, 5:5:50, 0.5:0.5:50, 0.05:0.05:50, 0.005:0.005:50, and 0.0005:0.0005:50 ng μL^–1^. Three biological replicates of each dilution were prepared. The regression equations and *R*^2^ coefficient of determination for Cq value versus the natural logarithm of the ratio of the various DNA indicated are shown on the charts. Data represent means ± standard deviation where visible.

We next investigated the detection efficiencies of Db ITS and Dd ITS primers against the DNA of *D. batatas* and *D. destruens* under different DNA mixing ratios by decreasing the DNA concentration of one species while keeping the DNA concentration of the other species constant. Both Db ITS and Dd ITS primers were capable of quantifying each of their target pathogen DNA down to a ratio of 1:10000 (Figure 3A). Db ITS amplified *D. batatas* DNA fragments from 13.9 (±0.3) to 30.2 (±2.0) cycles and Dd ITS yielded amplicons from 16.5 (±0.2) to 31.3 (±0.5) cycles in the range of 1:1 to 1:10000. On the other hand, quantification beyond 1:10000 was limited in both primer sets. There were no false positives for either Db ITS or Dd ITS primers against the mixed DNA.

We assessed the ability of the newly developed primers to quantify fungal DNA mixed with abundant sweet potato DNA. Db ITS and Dd ITS primers produced amplicons specific to target fungal pathogen DNA from a *D. batatas*: *D. destruens*: sweet potato DNA ratio of 1:1:1 to 1:1:10000 without false positives (Figure 3B). Recovery of both *D. batatas* and *D. destruens* DNA in a mixed sample was not achieved beyond the detection limit being 1:1:10000, resulting in quantification from 13.6 (±0.2) to 28.8 (±0.3) cycles by Db ITS and 16.7 (±0.3) to 31.4 (±0.4) cycles by Dd ITS. These results demonstrated that *D. batatas* and *D. destruens* DNA present in a background of sweet potato DNA was detectable by using Db ITS and Dd ITS primers.

### Evaluation of Naturally Infected Sweet Potato Plants in the Field

**FIGURE 4:**
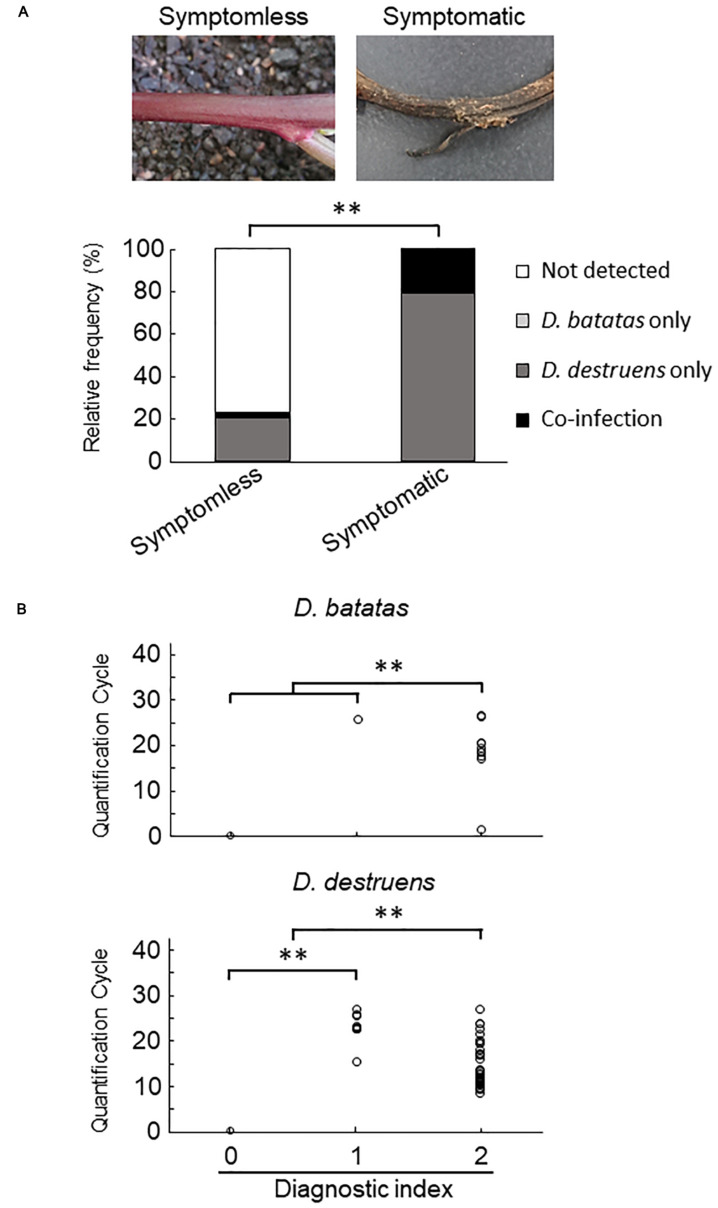
Frequency of amplification of *D. batatas* and *D. destruens* DNA from naturally infected sweet potato stems. **(A)** Relative frequency of *D. batatas* and *D. destruens* detection in sweet potato stems with or without symptomatic disorder. The relative frequency of detection of the two pathogens was significantly different between symptomless and symptomatic stem tissues (*n* = 77, *^∗∗^P* < 0.01, Fisher’s exact test). **(B)** Real-time PCR detection of *D. batatas* and *D. destruens* DNA from naturally infected sweet potato seedlings in the field. Quantification cycle using the new primers is graphed against diagnostic index of stem (0, symptomless with no pathogen detected; 1, symptomless but pathogen detected; and 2, symptomatic disorder with pathogen detected). The concentrations of *D. destruens* and *D. batatas* in stems with diagnostic index 2 were significantly higher than in those with index 0 or 1, and the concentration of *D. destruens* in stems with diagnostic index 1 was significantly higher than in those with index 0 (*n* = 77, *^∗∗^P* < 0.01, Steel–Dwass test).

**FIGURE 5:**
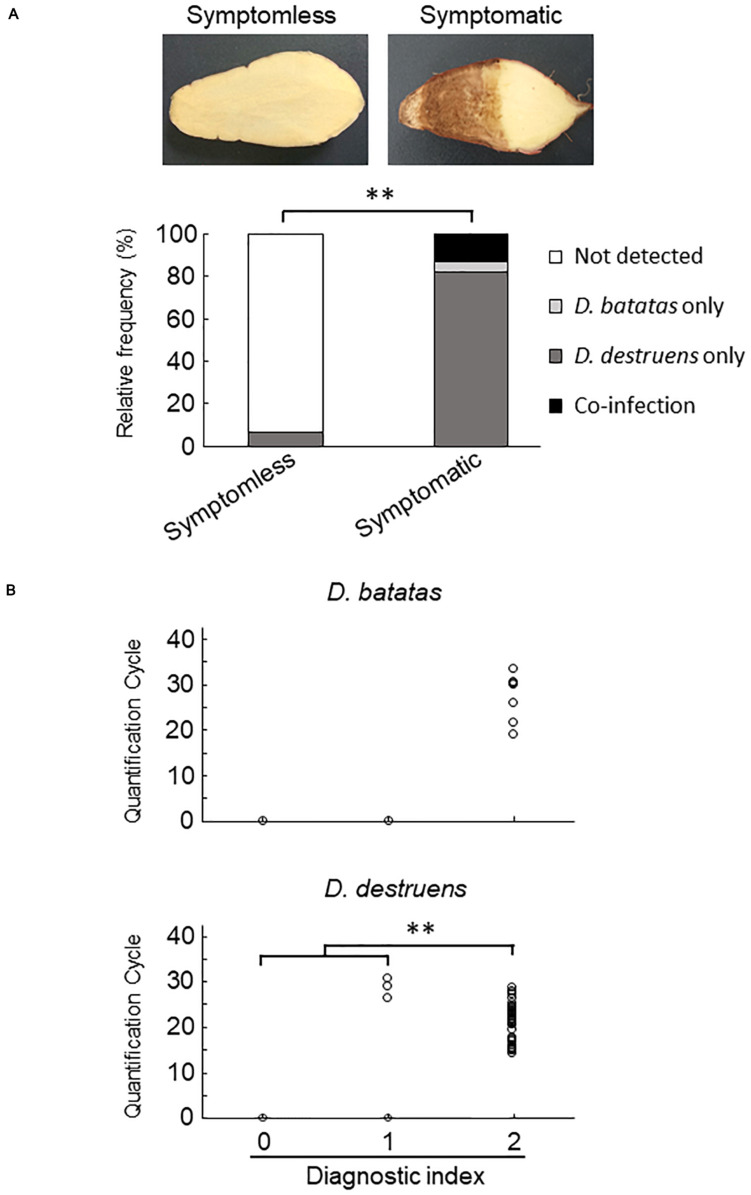
Frequency of amplification of *D. batatas* and *D. destruens* DNA from naturally infected sweet potato tubers. **(A)** Relative frequency of *D. batatas* and *D. destruens* detection in sweet potato tubers with or without symptomatic disorder. Detection frequency of the two pathogens among symptomless and symptomatic tuber tissues was highly differentiated (*n* = 83, *^∗∗^P* < 0.01, Fisher’s exact test). **(B)** Amplification of *D. batatas* and *D. destruens* DNA from naturally infected sweet potato tubers in the field. Diagnostic index of sweet potato tubers scored on a scale of 0 to 2 along with real-time PCR detection using the new primer sets (0, symptomless with no pathogen detected; 1, symptomless but pathogen detected; and 2, symptomatic disorder with pathogen detected). The concentration of *D. destruens* in tubers with diagnostic index 2 was significantly higher than in those with the index 1 (*n* = 83, *^∗∗^P* < 0.01, Steel–Dwass test), while there was no statistical significance in *D. batatas*.

We collected 55 sweet potato plants and 35 tubers with or without symptoms from 37 fields where foot rot disease appears in the Kyushu and Okinawa regions. The symptomatic specimens showed foot rot disease–like symptoms: i.e., black lesions with wilted tissues in the stems and discoloration with soft or rotten tissues in the tubers. From the collected seedlings and tubers, we generated stem samples (*N* = 77) and tuber samples (*N* = 83), respectively. For each sample, we applied a diagnostic index scored on a scale of 0 to 2 (see section “Materials and Methods”), based on the real-time PCR assay with the new primer sets. Overall, for both stem (Figure 4 and Supplementary Table 2) and tuber tissues (Figure 5 and Supplementary Table 3), *D. destruens* was found predominantly in symptomatic tissues rather than symptomless tissues; *D. batatas* was not as frequent as *D. destruens* in either symptomatic or symptomless tissues. The differences in relative frequency and concentration of *D. batatas* and *D. destruens* between symptomless and symptomatic sweet potato plants were highly significant in both stems and tubers (*P* < 0.01; Figures 4, 5). For symptomatic stem tissues (diagnostic index 2), specific amplicons of *D. destruens* were generated from all 38 samples: eight of these samples were also positive for *D. batatas*, indicating co-infection of *D. batatas* and *D. destruens* (Figure 4B). On the other hand, for symptomless stem tissues (diagnostic indexes 0 and 1), nine of 39 samples were positive for *D. destruens*, including one sample that displayed co-infection with *D. batatas*; one sample was positive for *D. batatas* alone. In symptomatic tuber tissues (diagnostic index 2), 37 out of 39 samples were positive for *D. destruens*, including five samples that displayed co-infection with *D. batatas*, and two samples were positive for *D. batatas* alone (Figure 5B). Specific amplicons of *D. destruens*, but not *D. batatas*, were also detected in three samples of symptomless tubers with pathogen (diagnostic index 1). The remaining 41 samples of symptomless tubers had no detectable pathogen (diagnostic index 0). In short, these results demonstrated that co-infection of *D. destruens* and *D. batatas* was detectable and distinguishable in real-time PCR diagnosis for foot rot disease.

## Discussion

This study demonstrates that co-infection of *D. batatas* and *D. destruens* in sweet potato can be detected and distinguished by real-time PCR using the new primer sets Db ITS and Dd ITS. The technical reliability of our new PCR-based method is expected to be sufficient for diagnostic use. Our finding that *D. destruens* is frequently presented in symptomless sweet potatoes as well as symptomatic plants indicates that, for practical diagnostic evaluation of field plants, it is necessary to assess the presence of *D. batatas* and *D. destruens* in stems and tubers of both symptomless and symptomatic tissues. Also, the findings highlight the potential risks of further distribution of the fungal pathogens through symptomless seedlings or seed potatoes. Further studies are required to establish a molecular-based diagnosis process for foot rot disease based on our real-time PCR technique. This process would be a powerful tool for field evaluation of foot rot disease in sweet potato, even in cases of co-infection with other soil-borne pathogens that have been difficult to diagnose until now.

To ensure the accuracy and reliability of our real-time PCR technique in different laboratories, it is important to evaluate various combinations of real-time PCR thermal cyclers and reagents (Borst et al., 2004; Schoder et al., 2005; Bacich et al., 2011). In addition, different approaches for DNA extraction may influence the sensitivity of PCR detection of *D. batatas* and *D. destruens*, as previously reported for several fungal pathogens (Fredricks et al., 2005; Kidd et al., 2020). We performed an interlaboratory test in four experienced PCR laboratories in our project, all of whom successfully applied the approach demonstrated in this study (K. Fujiwara, unpublished data). The detection sensitivity for *D. batatas* and *D. destruens* varied slightly in the interlaboratory studies when different real-time PCR reagents were used. Thus, it is necessary to optimize the PCR conditions and operation process when performing conventional PCR and real-time PCR for diagnosis targeting *D. batatas and D. destruens*.

In general, the specificity and quantitative nature of PCR amplification in detection of *D. batatas* and *D. destruens* is negatively impacted by the generation of false positives from a non-target DNA (Grosdidier et al., 2016). Our study demonstrates that our real-time PCR technique for distinguishing *D. batatas* and *D. destruens* was specific and generated no false positives for up to 32 cycles, which is within the detection limit of the primers. However, a potential drawback in that Db ITS and Dd ITS primers yield false positives to other closely-related *Diaporthe* and *Phomopsis* species (Rossman et al., 2014; Gao et al., 2016) might arise. Post-PCR approaches to screen for non-target amplification may need to be employed. For instance, in this study, amplicons generated from closely-related *Diaporthe* and *Phomopsis* species beyond the detection limit were distinguishable from *D. batatas* by DNA sequencing (K. Fujiwara, unpublished data). The evaluation of potential false positive results could also be achieved by other methods such as restriction fragment length polymorphism or microsatellite analysis (Koreth et al., 1996; Borst et al., 2004). Understanding the potential risks of obtaining false positives in PCR would allow field evaluation of foot rot disease in naturally infected sweet potato.

The use of real-time PCR assays may provide a new approach for managing foot rot disease of sweet potato. Currently, there are no chemicals that can be effectively used to control the disease (Kobayashi, 2019). However, the heavy use of fungicides is not advisable for sweet potato production because of food safety regulation in Japan. Also, development of fungicide-resistant *D. destruens* due to the heavy use of fungicides might be a major concern (Batzer and Mueller, 2020). In this study, *D. destruens* in symptomless sweet potato seedlings was frequently detected by real-time PCR using Dd ITS primers. Thus, our methodology can be used to screen for disease-free seedlings and prevent the use of infected seedlings or nurseries in the preparation of disease-free seedlings and seed tubers. Future use of the real-time PCR assays to monitor *D. destruens* in soil could potentially identify the risk of foot rot disease and guide control decisions before disease development. Thus, the PCR approach could contribute to the development of countermeasures and could help establish a scheme to control foot rot disease of sweet potato by evaluating seedlings and/or soils before planting, to identify fields where foot rot disease occurs.

## Conclusion

Our study provides a novel PCR method to identify *D. batatas*, which causes dry rot disease, and *D. destruens*, which causes foot rot disease, of sweet potato. The real-time PCR assay is specific for each of the fungal pathogens and can distinguish them in co-infected sweet potato. Currently, there is no effective cure or control measure for *D. destruens* in sweet potato production in Japan. We expect that our PCR assay will provide a useful tool for diagnosis of *D. destruens* and will form the foundation of the design of integrated disease management strategies for foot rot disease.

## Data Availability Statement

The original contributions presented in the study are included in the article/Supplementary Material, further inquiries can be directed to the corresponding author/s.

## Author Contributions

KF and HI conducted the experiments and drafted the manuscript. MU, MN, SK, and YN assisted in evaluating PCR results and provided critical advice on PCR. YOK, KN, YO, AK, AM, KH, and YK contributed to the collection of fungal isolates, field samples, and diagnosis of infected materials. YOK also contributed to the design and the management of a project including this work. All authors contributed to the article and approved the submitted version.

## Conflict of Interest

The authors declare one relevant Japanese patent application, 2020-140356.

## Footnotes

1 https://www.gene.affrc.go.jp/index_en.php

2 https://www.ncbi.nlm.nih.gov/tools/primer-blast/

3 https://blast.ncbi.nlm.nih.gov/Blast.cgi
