# Supplementary material for: Real-Time PCR Assay for the Diagnosis and Quantification of Co-infections by Diaporthe batatas and Diaporthe destruens in Sweet Potato
Source: Front Plant Sci. 2021 Jun 22;12:694053. doi: 10.3389/fpls.2021.694053 (PMC8258416; doi:10.3389/fpls.2021.694053)
Supplement: Supplementary file 1 [file Data_Sheet_1.zip › Supplementary Figure 1_caption.pdf]

SUPPLEMENTARY FIGURE 1. Sequences of forward and reverse primers for ITS1 and ITS2 of rDNA on the multiple-sequence alignment of the reference sequences of *Diaporthe batatas* and *Diaporthe desruens* strains. The DNA sequences of *D. batatas* (KU577616, MG827239, NR_152456) and *D. destruens* (JN848791, MH465671, MH465672, MH465673) were obtained from the NCBI database. Of 11 *D. batatas* isolates and 17 *D. destruens* isolates (Supplementary Table 1), the DNA sequences of *D. batatas* strain MOKM-3S-B and *D. destruens* strain KTJ-1R-a were used as a representative sequence.
