# Supplementary material for: Real-Time PCR Assay for the Diagnosis and Quantification of Co-infections by Diaporthe batatas and Diaporthe destruens in Sweet Potato
Source: Front Plant Sci. 2021 Jun 22;12:694053. doi: 10.3389/fpls.2021.694053 (PMC8258416; doi:10.3389/fpls.2021.694053)
Supplement: Supplementary file 1 [file Data_Sheet_1.zip › Supplementary Figure 2_caption.pdf]

SUPPLEMENTARY FIGURE 2. Specificity test of the newly-developed primers in conventional PCR.

Db ITS and Dd ITS primers were tested against closely related *Diaporthe* species and *Phomopsis* species as well as *D. batatas* and *D. destruens*. Template DNA was prepared from culture plates of each fungal species. A single PCR mixture was prepared by mixing three biological replicates of each of fungal pathogen DNA (5 ng μL^{-1}). No template was used for the negative control. PCR products were detected by 1% agarose gel electrophoresis. M, marker.
