# Supplementary material for: Real-Time PCR Assay for the Diagnosis and Quantification of Co-infections by Diaporthe batatas and Diaporthe destruens in Sweet Potato
Source: Front Plant Sci. 2021 Jun 22;12:694053. doi: 10.3389/fpls.2021.694053 (PMC8258416; doi:10.3389/fpls.2021.694053)
Supplement: Supplementary file 1 [file Data_Sheet_1.zip › Supplementary Figure 3_caption.pdf]

SUPPLEMENTARY FIGURE 3. Specificity test of the newly-developed primers in real-time PCR.

Db ITS and Dd ITS primers were tested against closely related *Diaporthe* species and *Phomopsis* species as well as *D. batatas* and *D. destruens*. Template DNA was prepared from culture plates of each fungal species. Real-time PCR reactions were prepared from three biological replicates of each of fungal pathogen DNA. Stem and tuberous DNA of healthy sweet potato plants as well as no template were used as negative controls. Data represent means ± standard deviation.
