# Supplementary material for: Real-Time PCR Assay for the Diagnosis and Quantification of Co-infections by Diaporthe batatas and Diaporthe destruens in Sweet Potato
Source: Front Plant Sci. 2021 Jun 22;12:694053. doi: 10.3389/fpls.2021.694053 (PMC8258416; doi:10.3389/fpls.2021.694053)
Supplement: Supplementary file 1 [file Data_Sheet_1.zip › Supplementary Table 1.docx]

**Supplementary Table 1. *Diaporthe batatas* and *Diaporthe destruens* isolates used in this study**

| **Accession No.** | **Isolate** | **Species** | **Isolation source** | **Location** |
| --- | --- | --- | --- | --- |
| LC543575 | MOKM-3S-B | *D. batatas* | stem | Miyazaki |
| LC543576 | MS-3RJ-A1 | *D. batatas* | storage root | Miyazaki |
| LC543577 | MS-1S-A | *D. batatas* | stem | Miyazaki |
| LC543578 | MT-2L-A1 | *D. batatas* | leaf | Miyazaki |
| LC543579 | MKM-1S1P-A | *D. batatas* | stem | Miyazaki |
| LC543580 | MKM-1S1P-B | *D. batatas* | stem | Miyazaki |
| LC543581 | A2 | *D. batatas* | stem | Kagoshima |
| LC543582 | A5 | *D. batatas* | stem | Kagoshima |
| LC543584 | A9 | *D. batatas* | stem | Kagoshima |
| LC543585 | A10 | *D. batatas* | stem | Kagoshima |
| LC543586 | A11 | *D. batatas* | stem | Kagoshima |
| LC543388 | GiN-1 | *D. destruens* | stem | Okinawa |
| LC543389 | KTS-S-a | *D. destruens* | stem | Kagoshima |
| LC543390 | KTS-S-d | *D. destruens* | stem | Kagoshima |
| LC543391 | KTJ-1R-a | *D. destruens* | storage root | Kagoshima |
| LC543392 | KTJ-5S-aR | *D. destruens* | stem | Kagoshima |
| LC543393 | KKO-R1-A | *D. destruens* | storage root | Kagoshima |
| LC543394 | MOKM-1SH-A2 | *D. destruens* | stem | Miyazaki |
| LC543565 | MZ-K121-1 | *D. destruens* | stem | Miyazaki |
| LC543566 | MZ-INO1-4 | *D. destruens* | storage root | Miyazaki |
| LC543567 | MZ-HKE2 | *D. destruens* | storage root | Miyazaki |
| LC543568 | MZ-F7 | *D. destruens* | stem | Miyazaki |
| LC543569 | MZ-F8 | *D. destruens* | stem | Miyazaki |
| LC543570 | MZ-F9 | *D. destruens* | stem | Miyazaki |
| LC543571 | A1 | *D. destruens* | stem | Kagoshima |
| LC543572 | A3 | *D. destruens* | stem | Kagoshima |
| LC543573 | A4 | *D. destruens* | stem | Kagoshima |
| LC543574 | A7 | *D. destruens* | stem | Kagoshima |
