# Supplementary material for: Real-Time PCR Assay for the Diagnosis and Quantification of Co-infections by Diaporthe batatas and Diaporthe destruens in Sweet Potato
Source: Front Plant Sci. 2021 Jun 22;12:694053. doi: 10.3389/fpls.2021.694053 (PMC8258416; doi:10.3389/fpls.2021.694053)
Supplement: Supplementary file 1 [file Data_Sheet_1.zip › Supplementary Table 2.docx]

**Supplementary Table 2.** Frequency of amplification of *D. destruens* and *D. batatas* from sweet potato stems from the field.

| Sample | Location | Source | Template DNA  (ng μL^-1^) | Diagnostic index^a^ | Cq value^b^ | |
| --- | --- | --- | --- | --- | --- | --- |
|  |  |  |  |  | *D. destruens* | *D. batatas* |
| 1 | Fukuoka | Stem | 84.1 | 2 | 8.9 | Undetermined |
| 2 | Fukuoka | Stem | 37.0 | 2 | 9.9 | Undetermined |
| 3 | Fukuoka | Stem | 64.9 | 2 | 11.7 | Undetermined |
| 4 | Fukuoka | Stem | 32.6 | 2 | 12.1 | Undetermined |
| 5 | Fukuoka | Stem | 78.9 | 2 | 19.3 | Undetermined |
| 6 | Fukuoka | Stem | 61.2 | 2 | 19.6 | Undetermined |
| 7 | Fukuoka | Stem | 38.0 | 2 | 20.6 | Undetermined |
| 8 | Fukuoka | Stem | 67.8 | 2 | 22.9 | Undetermined |
| 9 | Fukuoka | Stem | 108.0 | 0 | Undetermined | Undetermined |
| 10 | Fukuoka | Stem | 42.8 | 0 | Undetermined | Undetermined |
| 11 | Fukuoka | Stem | 31.6 | 0 | Undetermined | Undetermined |
| 12 | Fukuoka | Stem | 45.3 | 0 | Undetermined | Undetermined |
| 13 | Fukuoka | Stem | 44.7 | 0 | Undetermined | Undetermined |
| 14 | Fukuoka | Stem | 26.0 | 0 | Undetermined | Undetermined |
| 15 | Fukuoka | Stem | 72.6 | 0 | Undetermined | Undetermined |
| 16 | Fukuoka | Stem | 92.2 | 0 | Undetermined | Undetermined |
| 17 | Kagoshima | Stem | 55.0 | 2 | 9.8 | Undetermined |
| 18 | Kagoshima | Stem | 55.6 | 2 | 11.9 | Undetermined |
| 19 | Kagoshima | Stem | 31.4 | 2 | 11.9 | 19.0 |
| 20 | Kagoshima | Stem | 45.1 | 2 | 12.4 | Undetermined |
| 21 | Kagoshima | Stem | 53.1 | 2 | 12.8 | 20.6 |
| 22 | Kagoshima | Stem | 42.0 | 2 | 13.1 | Undetermined |
| 23 | Kagoshima | Stem | 19.1 | 2 | 13.1 | Undetermined |
| 24 | Kagoshima | Stem | 28.3 | 2 | 14.1 | Undetermined |
| 25 | Kagoshima | Stem | 56.2 | 2 | 19.0 | 21.8 |
| 26 | Kagoshima | Stem | 35.4 | 1 | 17.2 | Undetermined |
| 27 | Kagoshima | Stem | 40.7 | 1 | 25.9 | Undetermined |
| 28 | Kagoshima | Stem | 109.2 | 1 | 26.6 | 29.5 |
| 29 | Kagoshima | Stem | 40.6 | 0 | Undetermined | Undetermined |
| 30 | Kagoshima | Stem | 56.8 | 0 | Undetermined | Undetermined |
| 31 | Kagoshima | Stem | 116.7 | 0 | Undetermined | Undetermined |
| 32 | Kagoshima | Stem | 64.4 | 0 | Undetermined | Undetermined |
| 33 | Kumamoto | Stem | 35.0 | 2 | 11.1 | Undetermined |
| 34 | Kumamoto | Stem | 54.3 | 2 | 11.9 | Undetermined |
| 35 | Kumamoto | Stem | 35.0 | 2 | 12.2 | Undetermined |
| 36 | Kumamoto | Stem | 20.2 | 2 | 12.4 | 30.5 |
| 37 | Kumamoto | Stem | 46.5 | 2 | 12.4 | Undetermined |
| 38 | Kumamoto | Stem | 32.9 | 2 | 14.9 | Undetermined |
| 39 | Kumamoto | Stem | 17.5 | 2 | 14.9 | Undetermined |
| 40 | Kumamoto | Stem | 81.8 | 2 | 15.3 | Undetermined |
| 41 | Kumamoto | Stem | 24.7 | 2 | 19.1 | Undetermined |
| 42 | Kumamoto | Stem | 33.3 | 0 | Undetermined | Undetermined |
| 43 | Kumamoto | Stem | 21.6 | 0 | Undetermined | Undetermined |
| 44 | Kumamoto | Stem | 29.7 | 0 | Undetermined | Undetermined |
| 45 | Kumamoto | Stem | 36.2 | 0 | Undetermined | Undetermined |
| 46 | Kumamoto | Stem | 34.1 | 0 | Undetermined | Undetermined |
| 47 | Kumamoto | Stem | 75.0 | 2 | 22.1 | Undetermined |
| 48 | Kumamoto | Stem | 21.6 | 2 | 22.1 | 23.1 |
| 49 | Miyazaki | Stem | 32.7 | 2 | 23.1 | Undetermined |
| 50 | Miyazaki | Stem | 21.7 | 2 | 24.8 | Undetermined |
| 51 | Miyazaki | Stem | 25.6 | 2 | 27.4 | Undetermined |
| 52 | Miyazaki | Stem | 18.1 | 2 | 27.5 | Undetermined |
| 53 | Miyazaki | Stem | 24.0 | 1 | 26.1 | Undetermined |
| 54 | Miyazaki | Stem | 43.5 | 1 | 26.2 | Undetermined |
| 55 | Miyazaki | Stem | 70.7 | 1 | 26.8 | Undetermined |
| 56 | Miyazaki | Stem | 98.4 | 1 | 29.6 | Undetermined |
| 57 | Miyazaki | Stem | 74.2 | 1 | 30 | Undetermined |
| 58 | Miyazaki | Stem | 43.4 | 1 | 31.5 | Undetermined |
| 59 | Miyazaki | Stem | 50.0 | 0 | Undetermined | Undetermined |
| 60 | Miyazaki | Stem | 49.5 | 0 | Undetermined | Undetermined |
| 61 | Miyazaki | Stem | 43.0 | 0 | Undetermined | Undetermined |
| 62 | Miyazaki | Stem | 28.2 | 0 | Undetermined | Undetermined |
| 63 | Miyazaki | Stem | 38.4 | 0 | Undetermined | Undetermined |
| 64 | Miyazaki | Stem | 20.9 | 0 | Undetermined | Undetermined |
| 65 | Miyazaki | Stem | 32.2 | 0 | Undetermined | Undetermined |
| 66 | Miyazaki | Stem | 63.0 | 0 | Undetermined | Undetermined |
| 67 | Nagasaki | Stem | 66.7 | 2 | 10.1 | 20.6 |
| 68 | Nagasaki | Stem | 41.7 | 2 | 11.3 | 19.6 |
| 69 | Nagasaki | Stem | 72.1 | 1 | Undetermined | 30.1 |
| 70 | Nagasaki | Stem | 138.6 | 0 | Undetermined | Undetermined |
| 71 | Nagasaki | Stem | 36.3 | 0 | Undetermined | Undetermined |
| 72 | Nagasaki | Stem | 32.2 | 0 | Undetermined | Undetermined |
| 73 | Nagasaki | Stem | 82.0 | 0 | Undetermined | Undetermined |
| 74 | Okinawa | Stem | 26.8 | 2 | 13.9 | 23.00 |
| 75 | Okinawa | Stem | 13.6 | 2 | 18.1 | Undetermined |
| 76 | Okinawa | Stem | 14.1 | 2 | 25.9 | Undetermined |
| 77 | Okinawa | Stem | 27.3 | 2 | 31.2 | Undetermined |

^a^ Diagnostic index: 0, no symptoms with no pathogen detected; 1, symptomless but pathogen detected; 2, symptomatic disorder with pathogen detected

^b^ Cq (quantification cycle) values are plotted in Figure 4B
