# Supplementary material for: Real-Time PCR Assay for the Diagnosis and Quantification of Co-infections by Diaporthe batatas and Diaporthe destruens in Sweet Potato
Source: Front Plant Sci. 2021 Jun 22;12:694053. doi: 10.3389/fpls.2021.694053 (PMC8258416; doi:10.3389/fpls.2021.694053)
Supplement: Supplementary file 1 [file Data_Sheet_1.zip › Supplementary Table 3.docx]

**Supplementary Table 3.** Frequency of amplification of *D. destruens* and *D. batatas* from tubers in field.

| Sample | Location | Source | Template DNA  (ng μL^-1^) | Diagnostic index^a^ | Cq value^b^ | |
| --- | --- | --- | --- | --- | --- | --- |
|  |  |  |  |  | *D. destruens* | *D. batatas* |
| 1 | Fukuoka | Tuber | 22.3 | 2 | 13.3 | Undetermined |
| 2 | Fukuoka | Tuber | 5.91 | 2 | 14.2 | Undetermined |
| 3 | Fukuoka | Tuber | 18.6 | 2 | 14.5 | Undetermined |
| 4 | Fukuoka | Tuber | 19.7 | 2 | 15.2 | Undetermined |
| 5 | Fukuoka | Tuber | 106.0 | 2 | 21.6 | Undetermined |
| 6 | Fukuoka | Tuber | 60.4 | 2 | 21.9 | Undetermined |
| 7 | Fukuoka | Tuber | 45.0 | 0 | Undetermined | Undetermined |
| 8 | Fukuoka | Tuber | 121.2 | 0 | Undetermined | Undetermined |
| 9 | Fukuoka | Tuber | 34.7 | 0 | Undetermined | Undetermined |
| 10 | Kagoshima | Tuber | 20.7 | 2 | 18.7 | Undetermined |
| 11 | Kagoshima | Tuber | 23.1 | 2 | 18.7 | Undetermined |
| 12 | Kagoshima | Tuber | 64.2 | 2 | 19.1 | Undetermined |
| 13 | Kagoshima | Tuber | 74.0 | 2 | 19.5 | Undetermined |
| 14 | Kagoshima | Tuber | 65.1 | 2 | 19.8 | Undetermined |
| 15 | Kagoshima | Tuber | 64.2 | 2 | 20.2 | Undetermined |
| 16 | Kagoshima | Tuber | 36.9 | 2 | 20.3 | Undetermined |
| 17 | Kagoshima | Tuber | 27.7 | 2 | 21.4 | Undetermined |
| 18 | Kagoshima | Tuber | 17.1 | 2 | 24.3 | Undetermined |
| 19 | Kagoshima | Tuber | 21.6 | 0 | Undetermined | Undetermined |
| 20 | Kagoshima | Tuber | 51.2 | 0 | Undetermined | Undetermined |
| 21 | Kagoshima | Tuber | 62.5 | 0 | Undetermined | Undetermined |
| 22 | Kagoshima | Tuber | 55.1 | 0 | Undetermined | Undetermined |
| 23 | Kagoshima | Tuber | 69.3 | 0 | Undetermined | Undetermined |
| 24 | Kagoshima | Tuber | 17.9 | 0 | Undetermined | Undetermined |
| 25 | Kagoshima | Tuber | 11.0 | 0 | Undetermined | Undetermined |
| 26 | Kagoshima | Tuber | 48.1 | 0 | Undetermined | Undetermined |
| 27 | Kagoshima | Tuber | 65.2 | 0 | Undetermined | Undetermined |
| 28 | Kagoshima | Tuber | 25.0 | 0 | Undetermined | Undetermined |
| 29 | Kagoshima | Tuber | 44.7 | 0 | Undetermined | Undetermined |
| 30 | Kagoshima | Tuber | 38.2 | 0 | Undetermined | Undetermined |
| 31 | Kagoshima | Tuber | 25.7 | 0 | Undetermined | Undetermined |
| 32 | Kagoshima | Tuber | 32.9 | 0 | Undetermined | Undetermined |
| 33 | Kagoshima | Tuber | 47.9 | 0 | Undetermined | Undetermined |
| 34 | Kagoshima | Tuber | 33.5 | 0 | Undetermined | Undetermined |
| 35 | Kagoshima | Tuber | 46.6 | 0 | Undetermined | Undetermined |
| 36 | Kagoshima | Tuber | 65.2 | 0 | Undetermined | Undetermined |
| 37 | Kumamoto | Tuber | 15.1 | 2 | 11.9 | 30.262 |
| 38 | Kumamoto | Tuber | 36.7 | 2 | 12.2 | Undetermined |
| 39 | Kumamoto | Tuber | 10.8 | 2 | 12.4 | Undetermined |
| 40 | Kumamoto | Tuber | 27.2 | 2 | 12.4 | 30.828 |
| 41 | Kumamoto | Tuber | 28.4 | 2 | 14.9 | Undetermined |
| 42 | Kumamoto | Tuber | 18.9 | 2 | 23.1 | Undetermined |
| 43 | Kumamoto | Tuber | 43.5 | 2 | 27.4 | Undetermined |
| 44 | Kumamoto | Tuber | 54.6 | 2 | Undetermined | 30.6 |
| 45 | Kumamoto | Tuber | 12.5 | 2 | Undetermined | 34.4 |
| 46 | Kumamoto | Tuber | 40.3 | 1 | 29.0 | Undetermined |
| 47 | Kumamoto | Tuber | 18.0 | 0 | Undetermined | Undetermined |
| 48 | Kumamoto | Tuber | 38.1 | 0 | Undetermined | Undetermined |
| 49 | Kumamoto | Tuber | 16.0 | 0 | Undetermined | Undetermined |
| 50 | Kumamoto | Tuber | 17.6 | 0 | Undetermined | Undetermined |
| 51 | Kumamoto | Tuber | 55.8 | 0 | Undetermined | Undetermined |
| 52 | Kumamoto | Tuber | 31.9 | 0 | Undetermined | Undetermined |
| 53 | Kumamoto | Tuber | 27.4 | 0 | Undetermined | Undetermined |
| 54 | Kumamoto | Tuber | 30.8 | 0 | Undetermined | Undetermined |
| 55 | Kumamoto | Tuber | 53.5 | 0 | Undetermined | Undetermined |
| 56 | Kumamoto | Tuber | 21.4 | 0 | Undetermined | Undetermined |
| 57 | Miyazaki | Tuber | 15.1 | 2 | 11.2 | Undetermined |
| 58 | Miyazaki | Tuber | 36.7 | 2 | 20.3 | Undetermined |
| 59 | Miyazaki | Tuber | 10.8 | 2 | 20.9 | Undetermined |
| 60 | Miyazaki | Tuber | 27.3 | 2 | 22.5 | Undetermined |
| 61 | Miyazaki | Tuber | 28.4 | 2 | 23.2 | Undetermined |
| 62 | Miyazaki | Tuber | 18.9 | 2 | 23.2 | Undetermined |
| 63 | Miyazaki | Tuber | 43.5 | 2 | 23.8 | Undetermined |
| 64 | Miyazaki | Tuber | 54.6 | 2 | 25.7 | Undetermined |
| 65 | Miyazaki | Tuber | 12.5 | 2 | 26.7 | Undetermined |
| 66 | Miyazaki | Tuber | 40.3 | 1 | 25.8 | Undetermined |
| 67 | Miyazaki | Tuber | 18.0 | 1 | 31.1 | Undetermined |
| 68 | Miyazaki | Tuber | 38.1 | 0 | Undetermined | Undetermined |
| 69 | Miyazaki | Tuber | 16.0 | 0 | Undetermined | Undetermined |
| 70 | Miyazaki | Tuber | 17.6 | 0 | Undetermined | Undetermined |
| 71 | Miyazaki | Tuber | 55.8 | 0 | Undetermined | Undetermined |
| 72 | Miyazaki | Tuber | 32.0 | 0 | Undetermined | Undetermined |
| 73 | Miyazaki | Tuber | 27.4 | 0 | Undetermined | Undetermined |
| 74 | Miyazaki | Tuber | 30.8 | 0 | Undetermined | Undetermined |
| 75 | Nagasaki | Tuber | 20.3 | 2 | 11.7 | 20.1 |
| 76 | Nagasaki | Tuber | 10.6 | 2 | 17.5 | Undetermined |
| 77 | Nagasaki | Tuber | 47.1 | 2 | 22.3 | 25.1 |
| 78 | Nagasaki | Tuber | 28.2 | 2 | 27.4 | Undetermined |
| 79 | Nagasaki | Tuber | 30.6 | 2 | 28.5 | 16.8 |
| 80 | Nagasaki | Tuber | 91.7 | 0 | Undetermined | Undetermined |
| 81 | Nagasaki | Tuber | 30.8 | 0 | Undetermined | Undetermined |
| 82 | Nagasaki | Tuber | 19.3 | 0 | Undetermined | Undetermined |
| 83 | Okinawa | Tuber | 12.4 | 2 | 21.9 | Undetermined |

^a^ Diagnostic index: 0, no symptoms with no pathogen detected; 1, symptomless but pathogen detected; 2, symptomatic disorder with pathogen detected

^b^ Cq (quantification cycle) values are plotted in Figure 5B
