# Supplementary figures and images for: Real-Time PCR Assay for the Diagnosis and Quantification of Co-infections by Diaporthe batatas and Diaporthe destruens in Sweet Potato
Source: Front Plant Sci. 2021 Jun 22;12:694053. doi: 10.3389/fpls.2021.694053 (PMC8258416; doi:10.3389/fpls.2021.694053)

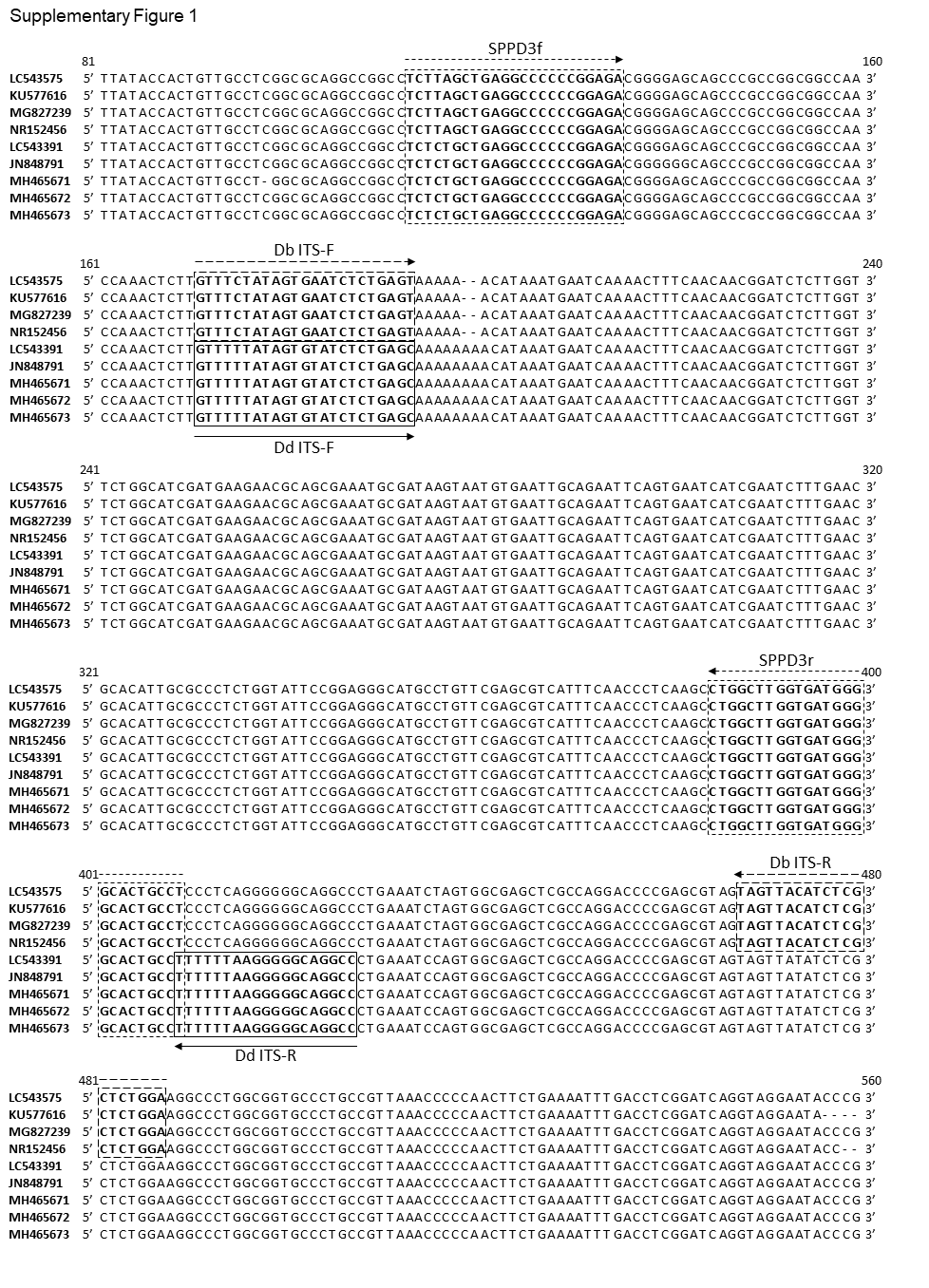

Supplement: Supplementary file 1 [file Data_Sheet_1.zip › Supplementary Figure 1.TIFF]

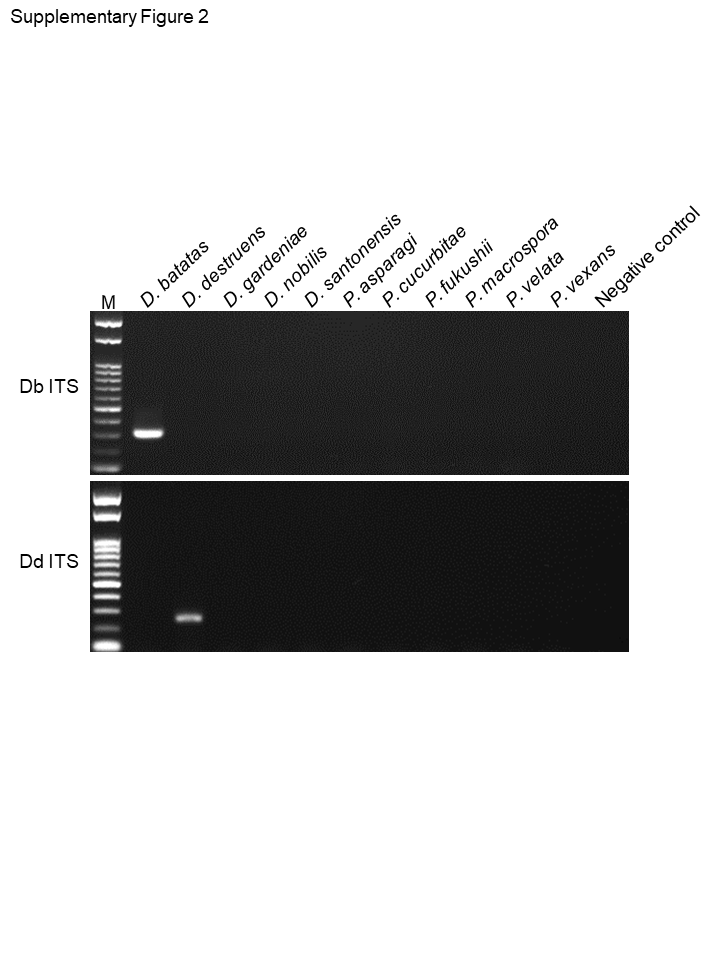

Supplement: Supplementary file 1 [file Data_Sheet_1.zip › Supplementary Figure 2.TIFF]

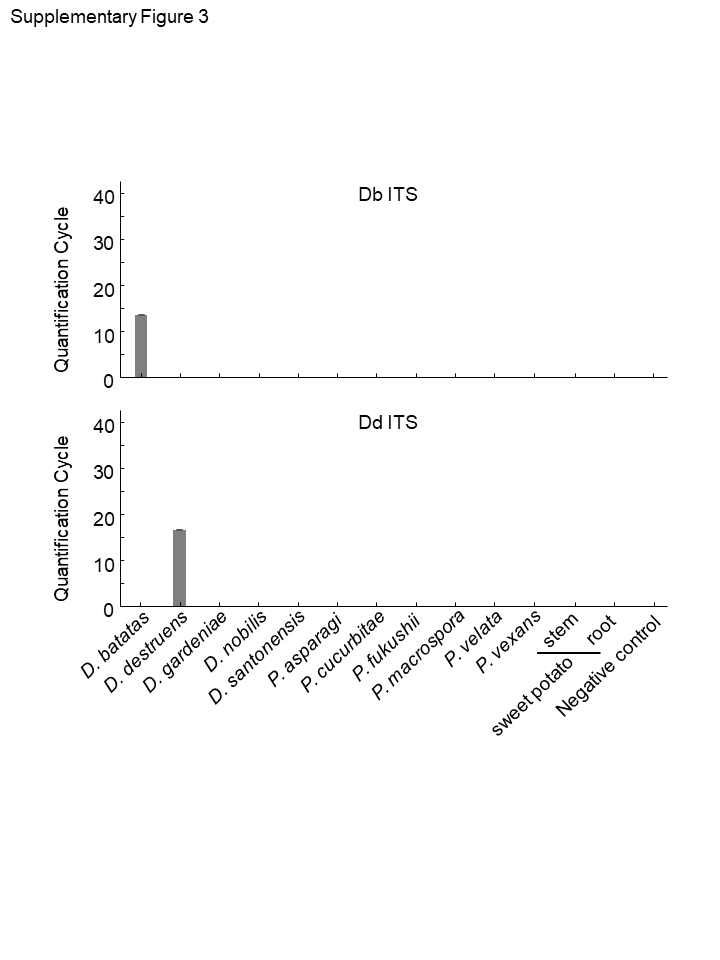

Supplement: Supplementary file 1 [file Data_Sheet_1.zip › Supplementary Figure 3.TIFF]
